# Two critical events in the development of the vascular system: pruning and remodeling

**DOI:** 10.1007/s10238-025-01720-0

**Published:** 2025-05-28

**Authors:** Domenico Ribatti

**Affiliations:** Department of Translational Biomedicine and Neuroscience, University of Bari, Bari, Italy

**Keywords:** Angiogenesis, Pruning, Remodeling, Vasculogenesis

## Abstract

The cardiovascular system is the first functional organ system to develop in the vertebrate embryo. The primitive heart and primitive vascular plexus are formed through vasculogenesis and thereafter through angiogenesis. After the establishment of new connections within the vascular network, a pruning and remodeling process occurs after which some branches are stabilized, whereas others regress. The aim of this article is to describe the most common pruning and remodeling forms, which take place during organogenesis in the experimental and human systems.

## The development of the cardiovascular system

The cardiovascular system is the first functional organ system to develop in the vertebrate embryo. The developing heart arises from lateral plate mesoderm early in embryogenesis and begins to emerge shortly after gastrulation. The primitive heart and primitive vascular plexus are formed through vasculogenesis, i.e., the formation of vessels from endothelial cells differentiating in situ from CD34^+^ mesodermal cells (angioblasts), and thereafter through angiogenesis, i.e., the new formation, sprouting of vessels from pre-existing ones [1–4]. Initiation of sprouting requires the specification of endothelial cells into tip and stalk cells bearing different morphologies and functional properties. Endothelial tip cells primarily migrate but proliferate only minimally, in contrast to endothelial stalk cells, which do proliferate [5]. Tip cells express delta‐like ligand 4 (DLL4), which binds Notch receptors on neighboring cells, inducing stalk cell formation. DLL4 signaling induces Notch‐intracellular domain (NICD) to be cleaved in stalk cells, which then reprograms the cell to produce vascular endothelial growth factor receptor1 (VEGFR1) instead of VEGFR2 [6]. When two adjacent tip cells come in contact, they can fuse and a lumenized, perfused vessel is formed. The leading tip cell responds to a VEGF gradient by migrating outward from the parent vessel up the gradient [7]. VEGF induces the formation and extension of filopodia as well as the expression of DLL4 in the tip cells, and filopodia engage with those of a nearby tip cell to form a ‘‘bridge’’ and the formation of a new vessel [8]. Upon maturation of the vessel, endothelial cells then regain a quiescent non-proliferative phenotype, corresponding to phalanx cells, with high VEGFR1 levels [9].

The dorsal aorta originates through vasculogenesis type I in which angioblasts arise at the site of vessel formation, whereas the endocardium, ventral aorta, and posterior cardinal veins form through vasculogenesis type II from angioblasts which originate at distant site and migrate as individual cells and small groups to the site of vessel formation [10]. Vasculogenesis is positively regulated by mesodermal-derived factors and negatively by ectodermal factors [11].

Further organogenesis during development is dependent on the delivery of oxygen and nutrients facilitated by a functional circulatory system, and major defects in the developing vasculature led to early embryonic lethality. Oxygen can be considered a repellent, while the lack of oxygen is a strong attractant for vessel sprouting. Hypoxia activates hypoxia-inducible transcription factors (HIFs) which turn on the expression of angiogenic genes such as VEGF, inducing vessels to branch toward the hypoxic tissue [12]. Thus, when HIF-dependent VEGF expression is genetically dysregulated, organs fail to grow and function normally, because of insufficient vessel branching and growth [13].

After the establishment of new connections within the vascular network, a pruning and remodeling process occurs after which some branches are stabilized, whereas others regress. Once mature, the vasculature consists of a system of arteries, arterioles, capillaries, venules, and veins that promotes the circulation of oxygenated blood between the heart, lung, and other organs.

## Pruning

Pruning was first described in the embryonic retina and involves the removal of supernumerary blood vessels from redundant channels [14]. The development of the retinal vasculature proceeds in two phases: formation of nascent vascular plexus by sprouting angiogenesis and remodeling of the nascent vascular plexus into a mature vascular tree displaying organization of larger trunks and smaller branches [15]. A critical role in this process is played by retinal astrocytes, which control endothelial growth and apoptosis, morphologically manifested as regulation of vascular pruning and capillary caliber [16]. In the retina, oxygen supply is a key factor in blood vessel pruning. Elevated oxygen levels induce vascular pruning [17]. Studies have highlighted the key role of oxygen and VEGF in the formation and remodeling of the retina vasculature [18, 19]. Hyperoxia suppresses VEGF production and leads to obliteration by apoptosis of vessels [20]. Vascular pruning is the initiating event in the pathogenesis of retinopathy of prematurity (ROP), a blindness-causing disease induced in premature infants placed in oxygen chambers, and VEGF protects retinal vessel from hyperoxia-induced obliteration in an experimental model of ROP [21].

Another model of vascular pruning is provided by modification of pulmonary vascularization under certain conditions, including chronic hypoxia, responsible for an increased total pulmonary vessel length, volume, endothelial surface area, and number of endothelial cells [22]. Moreover, chronic hypoxia leads to the development of increased pulmonary vascular resistance and pulmonary hypertension [23, 24]. The structural changes that are thought to underline the increased vascular resistance depend on remodeling of the walls of the pulmonary resistance vessels and pruning in the number of lung blood vessels [24, 25]. The changes include increased medial thickness of muscular arterioles, adventitial hypertrophy, and deposition of matrix components in the vascular walls [25, 26].

A structural alteration caused by chronic hypoxia is loss of small blood vessels [25, 27, 28]. Flow might affect vessel pruning, which, due to low wall shear stress, is highly sensitive to the pressure drop across a vascular network. In fact, the degree of pruning increases as the pressure drops increase [29]. Blood flow generally ceases in these excess capillaries, the lumina are obliterated, and the endothelial cell retracts toward adjacent capillaries. Recent advances have elucidated key mechano-transduction pathways, including Notch-DLL4 signaling, Piezo1 mechano-sensing, endothelial Krüppel-like factor 2 (KLF2) activation that regulate vessel stabilization or regression. Notch signaling is required for remodeling the primary plexus into the hierarchy of mature vascular beds and maintaining arterial fate and is essential for the homeostatic functions of differentiated arteries. Notch/DLL4 signaling stimulates vessel pruning by shifting the expression of vasoactive genes toward a vasoconstricting phenotype and inducing endothelial cell apoptosis [30, 31]. Inhibition of Notch/DLL4 pathway prevents retinal capillary regression in the mouse retina regulating vasoconstriction and blood flow [31]. One of the key roles of Piezo1 in the endothelium is the regulation of nitric oxide (NO) release, as mice with endothelial-specific Piezo1 deficiency lack flow-induced NO formation and vasodilation [32]. The expression and activation of Piezo1 correlate with repression of KLF2 transcript and protein levels [33]. Hear stress induces AKT signaling, resulting in KLF2 activation and upregulation of NO-synthase and superoxide dismutase, and consequent endothelial cell survival and NO-dependent vasodilation [33].

Intussusception may also contribute to pruning by formation of asymmetrically localized pillars at the branching points of larger vessels. As a result, daughter branches become partially obstructed or totally separated from the mother vessels [34]. This process hence was termed intussusceptive branching remodeling (IBR). IBR leads to modification of the branching geometry of supplying vessels, optimizing pre- and post-capillary flow properties, and leads to the removal of branches by pruning in response to changes in metabolic request.

## Remodeling

Developmental remodeling involves multiple regulated processes, including recruitment of mural cells, regulation of vascular calibers, and regression of excess microvessels. Remodeling angiogenesis refers to a wide array of changes in vascular architecture, including changes to existing vessels that range from expansion in vessel diameters, to regression and disappearance of vessels, fusion of smaller vessels to larger ones, and splitting of the larger vessels into smaller ones [35, 36]. These alterations are induced by changing flow of blood, with higher flow inducing expansion of vessels, while reduction of flow resulting in regression of vessels.

As the vascular system develops, the initial plexus becomes remodeled into a complex and heterogeneous array of blood vessels, including larger vessels such as arteries and veins, and smaller vessels such as venules, arterioles, and capillaries. Remodeling involves the growth of new vessels and the regression of others, as well as changes in the diameter of vessel lumen and vascular wall thickness. The developing vasculature responds dynamically to the growing needs of the embryo by remodeling vessels that is required to adapt to the changing hemodynamic and metabolic influences and to create a more efficient angioarchitecture. Some vessels may fuse to form a larger one, as occurs with fusion of the paired dorsal aortae, or they may establish new connections such as the coronary vessels that connect to the aorta [37]. It is likely that only a small number of embryonic blood vessels persist in adulthood [38], with most capillaries of the embryonic plexus regressing at some time in development to allow the differentiation of other tissues. The gross anatomy of the vascular system is characterized by highly reproducible branching patterns, with primary and secondary branches forming at precisely designed sites and with organ-specific architecture.

Angiogenesis in the corpus luteum of the cycling ovary, in which there are rapid changes in the vasculature, is illustrative of vascular remodeling [39]. Regression of the corpus luteum involves programmed cell death of endothelial cells and steroidogenic cells [40].

Other examples include the regression of capillaries in prechondrogenic regions to allow the differentiation of cartilage [41], the regression of the hyaloid vasculature to allow the development of the vitreous body in the eye [42] and the retinal vasculature, which undergoes dramatic vascular remodeling during the formation of the mature vasculature [43].

During embryological development, in the human vitreous the primary (vascular) vitreous is gradually replaced by the secondary (avascular) vitreous. With aging, the human vitreous undergoes slowly progressive remodeling, characterized by the gradual formation of collagenous condensations and liquefied spaces in the gel structure. The vitreal vasculature is a model of vascular remodeling, maturation, and regression. Formation and maturation of the vitreal vasculature involves initial vasculogenesis followed by angiogenesis, accompanied by pruning and remodeling of the maturing vitreal vessel network [44–47]. Tubedown-1 (tbdn-1) expression in endothelial cells is downregulated during the formation of capillary-like structures in vitro. Paradis et al. [48] showed that tbdn-1 was expressed highly in the vitreal vascular network during the pruning and remodeling phases, indicating that the developing vasculature responds to the growing needs of the embryo by remodeling vessels.

Vascular remodeling occurs during the formation of the aortic arches [49]. The major vessels that take blood away from the heart and deliver it to the arms and the head take their origin from the aortic arch and are derived from the arteries formed within the embryonic pharyngeal arches. These pharyngeal arch arteries, initially symmetrical, form in a cranial to caudal sequence within the pharyngeal mesenchyme and undergo a process of remodeling to produce the asymmetrical brachiocephalic arteries. It is important to note that the existence of the fifth pharyngeal arch artery remains controversial in human embryology. Despite the ever-increasing number of reported cases, convincing supportive developmental evidence remains elusive [50]. A complex interaction between the tissues of the pharyngeal arches and the genes they express is required to ensure that arterial formation and remodeling can proceed normally. In early development six paired pharyngeal arch arteries form sequentially to connect the two dorsal aortae to the truncal aortic sac. The first and the second pair of arches form and substantially regress, although they eventually contribute to the formation of the maxillary artery and the stapedial and hyoid arteries, respectively. The third, fourth, and sixth arches contribute to the common carotid, portions of the subclavian arteries, and the left aortic arch. The fifth arch is a subject of some debate, as it forms but regresses completely without forming functional vessels [50, 51]. The sixth arch gives rise to the left and right pulmonary arteries as well as the ductus arteriosus, which will regress steadily 1–3 days after birth. The coordinated pattern of regression leads to the proper formation of the carotid and subclavian arteries and a single left-sided aortic arch. Normally, the aortic sac divides into two horns: one on the right that develops brachiocephalic artery and the other on the left that makes the proximal segment of the aortic arch. The right horn makes the proximal segment of the right aortic arch, and the left horn is of unusually large caliber and is of abnormally long length. In addition to the extensive abnormal remodeling of the left brachiocephalic artery, the appearance of the dual ascending aortas due to persistence of the left ventral aorta between the origin of the sixth and the origin of the third aortic arch takes place.

If these regressions do not occur, or if regression occurs on the wrong side, many anomalies can occur, where vessels under arterial pressure impinge upon the bronchi and the esophagus, which can cause stridor, wheezing, esophageal obstruction, and failure to thrive [52, 53]. Aortic arch anomalies are common in patients with Di George syndrome [54].

By the fourth week of gestation in humans, a primitive venous system has developed, with paired left and right umbilical and vitelline veins draining into the sinus venosus at the base of the developing heart tube. Subsequently, the right vitelline vein regresses, whereas the left ones persist. Over the next 8 weeks, the entire right umbilical vein and the cranial portions of the left umbilical vein will regress, as the left umbilical vein is incorporated into portions of the vitelline vein. The umbilical vein, carrying oxygenated blood from the placenta, thus largely bypasses the liver to reach the newly formed inferior vena cava (IVC) via the ductus venosus [55]. The increasing venous flow from the developing organs of the gut directs the maintenance and expansion of a select hemodynamic path through the left and right vitelline veins and their connections. The superior and inferior mesenteric veins join the splenic, pancreatic, and duodenal veins to create the portal vein, which enters the liver. The portal vein promptly ramifies into the different hepatic lobes, delivering the products of these organs for processing and modification.

## Data Availability

No datasets were generated or analyzed during the current study.

## References

[1] Ribatti D. Genetic and epigenetic mechanisms in the early development of the vascular system. J Anat. 2006;208:139–52. 16441559 10.1111/j.1469-7580.2006.00522.x PMC2100191

[2] Ribatti D, Nico B, Crivellato E. Morphological and molecular aspects of physiological vascular morphogenesis. Angiogenesis. 2009;12:101–11. 19130273 10.1007/s10456-008-9125-1

[3] Risau W, Flamme I. Vasculogenesis. Ann Rev Cell Dev Biol. 1995;11:73–91. 8689573 10.1146/annurev.cb.11.110195.000445

[4] Risau W. Mechanisms of angiogenesis. Nature. 1997;386:671–4. 9109485 10.1038/386671a0

[5] Gerhardt H, Golding M, Fruttiger M, Ruhrberg C, Lundkvist A, Abramsson A, et al. VEGF guides angiogenic sprouting utilizing endothelial tip cell filopodia. J Cell Biol. 2003;161:1163–77. 12810700 10.1083/jcb.200302047 PMC2172999

[6] Phng LK, Gerhardt H. Angiogenesis: a team effort coordinated by notch. Dev Cell. 2009;16:196–208. 19217422 10.1016/j.devcel.2009.01.015

[7] Ruhrberg C, Gerhardt H, Golding M, Watson R, Ionnidou S, Frujisawa H, et al. Spatially restricted patterning cues provided by heparin-binding VEGF-A control blood vessel branching morphogenesis. Cancer Dev. 2002;16:2684–98. 10.1101/gad.242002 PMC187458 12381667

[8] Bentley K, Mariggi G, Gerhardt H, Bates PA. Tipping the balance: robustness of tip selection, migration and fusion in angiogenesis. PloS Comput Biol. 2009;5:e1000549. 19876379 10.1371/journal.pcbi.1000549 PMC2762315

[9] Ribatti D, Crivellato E. “Sprouting angiogenesis”. A reappraisal. Dev Biol. 2012;372:157–62. 23031691 10.1016/j.ydbio.2012.09.018

[10] Poole TJ, Coffin JD. Morphogenetic mechanisms in avian vascular development. In: Feinberg RN, Sherer GK, Auerbach R, editors. The development of the vascular system. Basel: Karger; 1991. p. 25–36.

[11] Augustine JM. Influence of the entoderm on mesodermal expansion in the area vasculosa of the chick. J Embryol Exp Morphol. 1991;65:89–103. 7334304

[12] Pugh CW, Ratcliffe PJ. Regulation of angiogenesis by hypoxia: role of the HIF system. Nat Med. 2003;9:677–84. 12778166 10.1038/nm0603-677

[13] Gerber HP, Hillan KJ, Ryan AM, Kowalski J, Keller GA, Rangell L, et al. VEGF is required for growth and survival in neonatal mice. Development. 1999;126:1149–59. 10021335 10.1242/dev.126.6.1149

[14] Ashton N. Oxygen and the growth and development of retinal vessels. Am J Ophthalmol. 1996;62:412–35. 10.1016/0002-9394(66)91322-5 5950745

[15] Fruttiger M. Development of the retinal vasculature. Angiogenesis. 2007;10:77–88. 17322966 10.1007/s10456-007-9065-1

[16] Duan LJ, Fong GH. Developmental vascular pruning in neonatal mouse retinas is programmed by the astrocytic oxygen-sensing mechanism. Development. 2019;146:dev175117. 30910827 10.1242/dev.175117 PMC6503987

[17] Claxton S, Fruttiger M. Oxygen modifies artery differentiation and network morphogenesis in the retinal vasculature. Dev Dyn. 2005;233:822–8. 15895398 10.1002/dvdy.20407

[18] Dorrell MI, Friedlander M. Mechanisms of endothelial cell guidance and vascular patterning in the developing mouse retina. Progr Ret Eye Res. 2006;25:277–95. 10.1016/j.preteyeres.2006.01.001 16515881

[19] Stone J, Maslim J. Mechanisms of retinal angiogenesis. Progr Retinal Eye Res. 1997;16:157–81.

[20] Stone J, Itin A, Alon T, Pe’er J, Gnessin H, Chan Ling T, et al. Development of retinal vasculature is mediated by hypoxia-induced vascular endothelial growth factor (VEGF) expression by neuroglia. J Neurosci. 1995;15:4783–7. 10.1523/JNEUROSCI.15-07-04738.1995 PMC6577882 7623107

[21] Alon T, Hem I, Itin A, Pe’er A, Stone J, Keshet E. Vascular endothelial growth factor acts as a survival factor for newly formed retinal vessels and has implications for retinopathy of prematurity. Nat Med. 1995;1:1024–208. 7489357 10.1038/nm1095-1024

[22] Howell K, Preston RJ, McLoughlin P. Chronic hypoxia causes angiogenesis in addition to remodeling in the adult pulmonary circulation. J Physiol. 2003;547:133–45. 12562951 10.1113/jphysiol.2002.030676 PMC2342608

[23] Hopkins N, McLoughlin P. The structural basis of pulmonary hypertension in chronic lung disease: remodeling, rarefaction or angiogenesis. J Anat. 2002;201:335–48. 12430958 10.1046/j.1469-7580.2002.00096.x PMC1570922

[24] Meyrick B, Reid L. The effect of continued hypoxia on rat pulmonary arterial circulation. An ultrastructural study. Lab Invest. 1978;38:188–200. 146763

[25] Rabinovitch M, Gamble W, Nadas AS, Miettinen OS, Reid L. Rat pulmonary circulation after chronic hypoxia: hemodynamic and structural features. Am J Physiol. 1979;236:H818–27. 443445 10.1152/ajpheart.1979.236.6.H818

[26] Stenmark KR, Mecham RP. Cellular and molecular mechanisms of pulmonary vascular remodeling. Annu Rev Physiol. 1997;59:89–144. 9074758 10.1146/annurev.physiol.59.1.89

[27] Hislop A, Reid L. New findings in pulmonary arteries of rats with hypoxia induced pulmonary hypertension. Br J Exp Pathol. 1976;57:542–54. 136978 PMC2041230

[28] Hislop A, Reid L. Changes in the pulmonary arteries of the rat during recovery from hypoxia-induced pulmonary hypertension. Br J Exp Pathol. 1977;58:653–62. 147098 PMC2041298

[29] Skalak TC, Price RJ. The role of mechanical stresses in microvascular remodeling. Microcirc. 1996;3:143–65. 10.3109/10739689609148284 8839437

[30] Lobov IB, Renard RA, Papadopoulos N, Gale NW, Thurston G, Yancopoulos GD, et al. Delta-like ligand 4 (Dll4) is induced by VEGF as a negative regulator of angiogenic sprouting. Proc Natl Acad Sci USA. 2007;104:3219–24. 17296940 10.1073/pnas.0611206104 PMC1805530

[31] Lobov IB, Cheung E, Wudali R, Cao J, Halasz G, Wei Y, et al. The Dll4/Notch pathway controls postangiogenic blood vessel remodeling and regression by modulating vasoconstriction and blood flow. Blood. 2011;117:6728–37. 21498671 10.1182/blood-2010-08-302067

[32] Wang S, Chennupati R, Kaur H, Iring A, Wettschureck N, Offermanns S. Endothelial cation channel PIEZO1 controls blood pressure by mediating flow-induced ATP release. J Clin Invest. 2016;126:4527–36. 27797339 10.1172/JCI87343 PMC5127677

[33] Dekker RJ, van Thienen JV, Rohlena J, de Jager SC, Elderkamp YW, et al. Endothelial KLF2 links local arterial shear stress levels to the expression of vascular tone-regulating genes. Am J Pathol. 2005;167:609–18. 16049344 10.1016/S0002-9440(10)63002-7 PMC1603569

[34] Djonov V, Kurz HM, Burri PH. Optimality in the developing vascular system: branching remodeling by means of intussusceptive as an efficient adaptation mechanism. Dev Dyn. 2002;224:391–402. 12203731 10.1002/dvdy.10119

[35] Baldwin HS. Early embryonic vascular development. Cardiovasc Res. 1996;31:E34–43. 8681344

[36] Wilting J, Christ B. Embryonic angiogenesis. A review. Naturwissenchaften. 1996;83:153–64. 10.1007/BF01143056 8643122

[37] Bogers AJ, Gittenberger-de Groot AC, Poelmann RE, Peault BM, Huysmans HA. Development of the origin of the coronary arteries, a matter of ingrowth or outgrowth? Anat Embryol. 1989;180:437–41. 10.1007/BF00305118 2619086

[38] Risau W. Differentiation of the endothelium. FASEB J. 1995;9:926–33. 7615161

[39] Augustin HG, Braun K, Telemenakis I, Modlich U, Kuhn W. Ovarian angiogenesis. Phenotypic characterization of endothelial cells in a physiological model of blood vessel growth and regression. Am J Pathol. 1995;147:339–51. 7543733 PMC1869809

[40] Augustin HG. Vascular morphogenesis in the ovary. Baillieres Best Pract Res Clin Obstet Gynaecol. 2000;14:867–82. 11141338 10.1053/beog.2000.0132

[41] Hallmann R, Feinberg RN, Latker CH, Sasse J, Risau W. Regression of blood vessels precedes cartilage differentiation during chick limb development. Differentiation. 1987;34:98–105. 3622953 10.1111/j.1432-0436.1987.tb00055.x

[42] Latker CH, Kuwubara T. Regression of the tunica vasculosa lentis in the postnatal rat. Invest Ophthalomol Vis Sci. 1981;21:689–99. 7298273

[43] Benjamin LE, Hemo I, Keshet E. A plasticity window for blood vessel remodeling is defined by pericyte coverage of the preformed endothelial network and is regulated by PDGF-B and VEGF. Development. 1998;125:1591–8. 9521897 10.1242/dev.125.9.1591

[44] Ash JD, Overbeek PA. Lens-specific VEGF-A expression induces angioblast migration and proliferation and stimulates angiogenic remodeling. Dev Biol. 2000;223:383–98. 10882523 10.1006/dbio.2000.9755

[45] Lang RA, Bishop JM. Macrophages are required for cell death and tissue remodeling in the developing mouse eye. Cell. 1993;74:453–62. 8348612 10.1016/0092-8674(93)80047-i

[46] Mitchell CA, Risau W, Drexler HC. Regression of vessels in the tunica vasculosa lentis is initiated by coordinated endothelial apoptosis: a role for vascular endothelial growth factor as a survival factor for endothelium. Dev Dyn. 1998;213:322–33. 9825867 10.1002/(SICI)1097-0177(199811)213:3<322::AID-AJA8>3.0.CO;2-E

[47] Yang K, Cepko CL. Flk-1, a receptor for vascular endothelial growth factor (VEGF), is expressed by retinal progenitor cells. J Neurosci. 1996;16:6089–99. 8815891 10.1523/JNEUROSCI.16-19-06089.1996 PMC6579183

[48] Paradis H, Liu CY, Saika S, Azhar M, Doetschman T, Good WV, et al. Tubedown-1 in remodeling of the developing vitreal vasculature in vivo and regulation of capillary outgrowth in vitro. Dev Biol. 2002;249:140–55. 12217325 10.1006/dbio.2002.0757

[49] Rosenquist GC. Aortic arches in the chick embryo: origin of the cells as determined by radioautoragiography mapping. Anat Rec. 1970;168:351–9. 5477181 10.1002/ar.1091680303

[50] Gupta SK, Gulati GS, Anderson RH. Clarifying the anatomy of the fifth arch artery. Ann Pediatr Cardiol. 2016;9:62–7. 27011696 10.4103/0974-2069.171392 PMC4782472

[51] Gupta SK, Bamforth SD, Anderson RH. How frequent is the fifth arch artery? Cardiol Young. 2015;25:628–46. 25351107 10.1017/S1047951114002182

[52] Kocis KC, Midgley FM, Ruckman RN. Aortic arch complex anomalies: 20-year experience with symptoms, diagnosis, associated cardiac defects, and surgical repair. Pediatr Cardiol. 1997;18:127–32. 9049126 10.1007/s002469900130

[53] Schmidt AMS, Larsen SH, Hjortdal VE. Vascular ring: early and long-term mortality and morbidity after surgical repair. J Pediatr Surg. 2018;53:1976–9. 29402450 10.1016/j.jpedsurg.2017.12.022

[54] Momma K, Matsuoka R, Takao A. Aortic arch anomalies associated with chromosome 22q11 deletion (CATCH 22). Pediatr Cardiol. 1999;20:97–102. 9986884 10.1007/s002469900414

[55] Collardeau-Frachon S, Scoazec JY. Vascular development and differentiation during human liver organogenesis. Anat Rec (Hoboken). 2008;291:614–27. 18484606 10.1002/ar.20679

